# Optical Monitoring in Neonatal Seizures

**DOI:** 10.3390/cells11162602

**Published:** 2022-08-21

**Authors:** Rachel Howard, Runci Li, Kelly Harvey-Jones, Vinita Verma, Frédéric Lange, Geraldine Boylan, Ilias Tachtsidis, Subhabrata Mitra

**Affiliations:** 1 Institute for Women’s Health, University College London, London WC1E 6BT, UK; 2 Medical Physics and Biomedical Engineering, University College London, London WC1E 6BT, UK; 3 INFANT Research Centre, University College Cork, T12 K8AF Cork, Ireland; 4 Department of Paediatrics and Child Health, Cork University Hospital, T12 DC4A Cork, Ireland

**Keywords:** neonatal seizures, near infrared spectroscopy, cerebral oxygenation, cerebral haemodynamics, cerebral metabolism

## Abstract

Background: Neonatal seizures remain a significant cause of morbidity and mortality worldwide. The past decade has resulted in substantial progress in seizure detection and understanding the impact seizures have on the developing brain. Optical monitoring such as cerebral near-infrared spectroscopy (NIRS) and broadband NIRS can provide non-invasive continuous real-time monitoring of the changes in brain metabolism and haemodynamics. Aim: To perform a systematic review of optical biomarkers to identify changes in cerebral haemodynamics and metabolism during the pre-ictal, ictal, and post-ictal phases of neonatal seizures. Method: A systematic search was performed in eight databases. The search combined the three broad categories: (neonates) AND (NIRS) AND (seizures) using the stepwise approach following PRISMA guidance. Results: Fifteen papers described the haemodynamic and/or metabolic changes observed with NIRS during neonatal seizures. No randomised controlled trials were identified during the search. Studies reported various changes occurring in the pre-ictal, ictal, and post-ictal phases of seizures. Conclusion: Clear changes in cerebral haemodynamics and metabolism were noted during the pre-ictal, ictal, and post-ictal phases of seizures in neonates. Further studies are necessary to determine whether NIRS-based methods can be used at the cot-side to provide clear pathophysiological data in real-time during neonatal seizures.

## 1. Introduction

Seizures are the most common neurological emergency in the neonatal period and require neonatal intensive care support [1]. The incidence of neonatal seizures varies between 1–5 per 1000 live births [2,3], with significantly higher rates in premature infants (57–132 per 1000 live births) [4,5]. Due to the predominance of sub-clinical or electrographic-only neonatal seizures, the use of electroencephalography (EEG) or amplitude-integrated electroencephalography (aEEG) is required for increased diagnostic accuracy. Electrographic neonatal seizures are commonly defined as sudden, repetitive, evolving, and stereotyped episodes of abnormal electrographic activity with a minimum duration of ten seconds [6]. Recently, the ten second duration was removed from the definition due to its arbitrary nature. The duration must now be sufficient to recognise the frequency, evolution, and morphology of the discharges [7].

Neonatal seizures are often the first clinical sign of underlying neurological dysfunction [8], with the most common causes being hypoxic-ischemic encephalopathy (HIE), perinatal stroke and intracranial haemorrhage [7]. Due to the frequency of these conditions and the relative excitability of the newborn brain, the neonatal period, in particular the first week of life, is the most vulnerable period for the development of seizures [5]. Exaggerated excitation is, however, critical for many developmental processes including the formation of synapses, cell differentiation and migration, and neurogenesis [9,10,11,12]. Disruption of the balance between excitation and inhibition can have a profound impact on neuro-developmental outcomes [9]. Recurrent and prolonged seizures disrupt the construction of cortical networks, adversely affect learning and memory, and are associated with further brain injury [13,14]. A substantial number of studies have now demonstrated that long-term structural and behavioural changes may occur following recurrent seizures [15,16,17,18,19,20,21,22,23,24,25], including seizures seen in HIE [26,27].

EEG remains the gold standard monitoring technique for the detection of neonatal seizures since many neonatal seizures do not have a clinical signature [7]. Both electrographic-only and electroclinical seizures have significant impacts on neurological outcomes [23,28,29,30,31,32]. When considering electrographic-only seizures in at-risk neonates, McBride et al. [28] noted a correlation between the occurrence of electrographic seizures and failure to thrive, severe cerebral palsy and an increased risk of death. Such results support the increasing amounts of evidence that electrographic seizures are associated with high morbidity and mortality rates in neonates [33]. Electrographic seizures have been demonstrated to increase the risk of subsequent epilepsy [34] and are associated with an increased amount of brain injury visualised on MRI [35]. Several pre-clinical studies have demonstrated that neonatal seizures can lead to long-term impaired learning, memory, and behaviour [36,37,38]. In term infants with HIE, outcomes at 18 months in the CoolCap trial were improved in neonates without seizures compared to those with [39].

There is now an increasing amount of evidence that adverse outcomes are associated with higher grades of HIE and an increased seizure burden [40]. The odds of a poor outcome increased over nine-fold (odds ratio 9.56) if a neonate had a total seizure burden of more than 40 min (*p* = 0.001), and eight-fold (OR: 8.00) if a neonate had a maximum hourly seizure burden of more than 13 min per hour (*p* = 0.003) [30]. Seizures at any stage were associated with higher rates of poor outcome (*p* = 0.003) at 2 years in a study with continuous video EEG monitoring following neonatal encephalopathy [41]. Glass et al. [26] demonstrated that by age four, the children that had had severe clinical seizures as an infant had worse full-scale intelligence quotients on the Wechsler Preschool and Primary Scale of Intelligence - Revised scale when compared to their peers.

Magnetic resonance spectroscopy (both ^1^H MRS and ^31^P MRS) has been used to assess the impact of neonatal seizures [42,43]. During seizures, an increase in metabolic activity resulting in an increase in oxygen consumption, glucose consumption and cerebral blood flow is expected. The increased supply of glucose and oxygen to the brain results in a decrease in brain energy metabolites [44]. When observing energy changes using ^31^P MRS during neonatal seizures, mitochondrial oxidative phosphorylation increased and high energy phosphates decreased [45], suggesting a decreased energy state during the ictal-period [46]. The disruption in cerebral oxidative metabolism can also be identified with MRS in infants with HIE [47,48]. Following neonatal HIE, disruption to cerebral haemodynamics and metabolism has been noted to evolve over time [49,50,51].

Near infrared spectroscopy (NIRS) was first introduced for optical monitoring by Jobsis in 1977 [52] and is attracting increasing amounts of attention in neonatology [53] due to its non-invasive nature, low cost and availability at the cot-side [54]. It is increasingly used to assess oxygenation in brain tissue as well as cerebral haemodynamics in real-time using light absorption by chromophores (particularly haemoglobin) in the near-infrared spectrum (650–900 nm). Commercial NIRS systems focus mainly on the quantification of haemoglobin concentration, and hence, measurements of oxygenation and haemodynamics. Recently, broadband NIRS (BNIRS), described as a technological extension to NIRS, has allowed interrogation of the full NIR spectrum along with the quantification of other chromophores in the brain tissue including cytochrome c oxidase (CCO), and wate.

In the literature, a variety of nomenclature is used to describe the various NIRS markers: oxyhaemoglobin (HbO, HbO_2_) and deoxyhaemoglobin (HHb, HbR) concentration changes. NIRS devices such as cerebral oximeters can derive the absolute tissue oxygen saturation as a percentage, which has been labelled as regional cerebral oxygenation (rSO_2_, rScO_2_), tissue oxygenation index (TOI) or tissue oxygen saturation (StO_2_, SctO_2_). For consistency, the following terminology will be used in the remainder of this paper: oxyhaemoglobin (HbO_2_), deoxyhaemoglobin (HHb) and cerebral tissue oxygenation (rSO_2_). Total haemoglobin (HbT, calculated as HbO_2_ + HHb), and haemoglobin difference (HbD, calculated as HbO_2_ – HHb) serve as representative markers of cerebral blood volume (CBV) and cerebral oxygen delivery, respectively. In previous studies, HbD has also proven to be a proxy marker of cerebral blood flow (CBF) assuming oxygen consumption does not change [55,56]. NIRS measurements are not pulse-synchronized like pulse oximetry monitors, and, therefore, provide a regionalized composite measure of oxygenation in venous, arterial, and capillary beds [57]. Cerebral tissue oxygenation mostly indicates venous oxygenation.

Fractional tissue oxygen extraction (FTOE) has been used to demonstrate metabolic changes in the brain and is calculated using the combined measurement of rSO_2_ from cerebral oximeters and peripheral arterial oxygen saturation from pulse oximeters (SaO2) (FTOE = SaO_2_ − rSO_2_/SaO_2_). It represents the balance between oxygen delivery and consumption. FTOE is, therefore, an indirect measure of mitochondrial oxidation but can be measured from a commercial NIRS system. The need to measure cerebral metabolism directly resulted in further developments in broadband (multiwavelength) NIRS (BNIRS) [58]. Compared to commercial NIRS systems that use only limited NIR wavelengths, BNIRS uses hundreds of NIR wavelengths to obtain complete NIR spectra of the brain tissue. This allows quantification of the oxidation state of the cytochrome-c-oxidase (oxCCO) concentration changes. CCO is located on the inner mitochondrial membrane in all mitochondria and is an integral transmembrane protein [59]. In the electron transport chain, it is the terminal electron acceptor responsible for approximately 95% of oxygen metabolism [60]. This enzyme is essential to generate adenosine triphosphate (ATP) efficiently during aerobic respiration. Since the concentration of CCO is assumed to be constant, the changes in oxCCO measured by NIRS are indicative of its redox state and, therefore, the utilization of oxygen in the surrounding tissue [61,62]. oxCCO can, therefore, be used reliably as a metabolic biomarker in the brain tissue.

In newborn infants, NIRS is considered to be an ideal neuromonitoring technique, as the thin skin and skull of the newborn allows deep penetration of light through to the brain tissue [63]. With evidence now emerging that NIRS monitoring is useful following both neonatal encephalopathy and for preterm brain monitoring, a systematic review is timely to identify the changes in optical markers related to neonatal seizures that can be monitored at the cot side. This systematic review summarises the changes in optical markers that have been described to date in studies of neonatal seizures that help improve our understanding of the pathophysiological changes that occur.

## 2. Materials and Methods

To identify eligible studies, the Preferred Items for Systematic Reviews and Meta-analysis (PRISMA) statement was used for guidance [64]. Details of the protocol for this systematic review were registered on PROSPERO to ensure a complete and transparent review was undertaken (registration CRD42021243178) [65].

An extensive search was undertaken using the following databases: Ovid MEDLINE(R), Embase Classic + Embase, Emcare, Maternity & Infant Care Database (MIDIRS), Web of Science, Scopus, PsycInfo, CINAHL plus and Cochrane library. The databases were searched individually to minimise the risk of missing key papers on 6 June 2022. Conferences were also monitored for any emerging research on the topic. Articles were filtered by language, selecting English only. There were three broad concepts used: category (neonates) AND monitoring (NIRS) AND subject (seizures). The search strategy for this review is presented in Table 1.

Publications were included in this review if they presented original data evaluating the use of NIRS in term or near-term newborn infants or infant animals reported to have had seizures. Study titles and abstracts were imported into Rayyan [66], a widely used online software that allows comprehensive documentation of selection decisions, to assist with traceability in the screening process before full-text assessments were undertaken. The first two authors independently screened the articles against the inclusion criteria. Articles grouped into “maybe” were discussed by the authors to assess their eligibility for inclusion. Articles were excluded if there was an abstract-only publication, and no full-text journal article available.

Traditionally, systematic reviews have excluded case reports; however, in cases where randomised controlled trials are not present in the literature, case reports can greatly contribute to the evidence base [67]. All study types were, therefore, included in this systematic review due to the lack of high-quality experimental designs available on this topic. To assess the quality of each study type adequately, a reputable quality assessment tool was selected for each study type included in the review. Included studies were critically appraised using the Newcastle-Ottawa Scale (NOS) [68] and the Joanna Briggs Institute (JBI) Critical Appraisal tool [69].

## 3. Results

**Figure 1:**
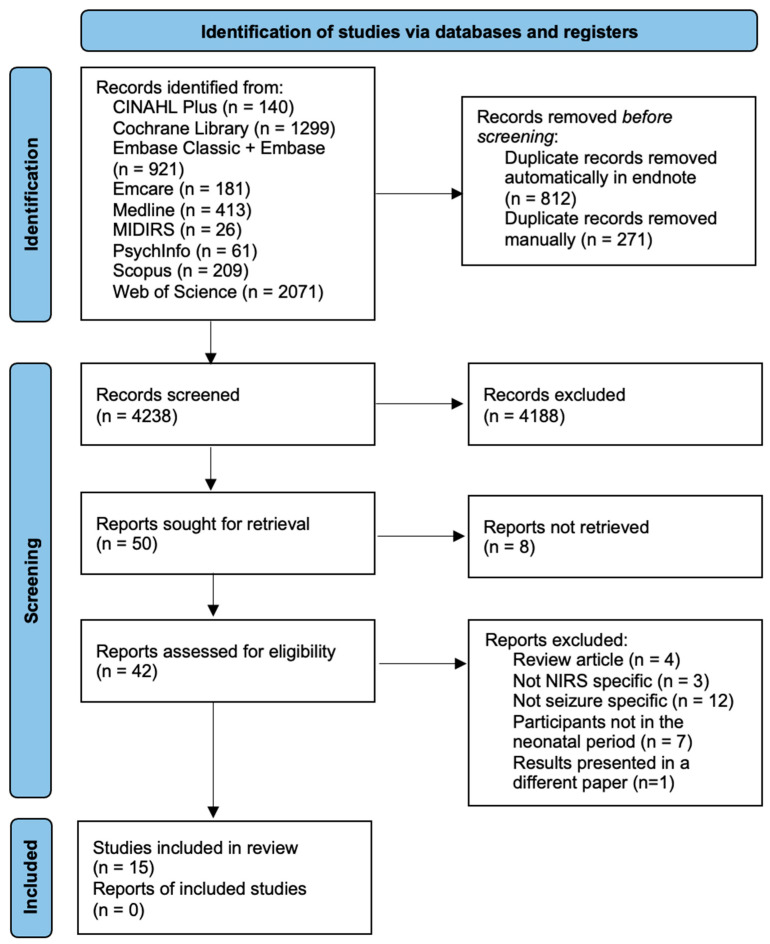
PRISMA flow chart showing identification of studies.

The initial search identified 5321 papers; following deduplication, 4238 articles were screened by title and abstract. Fifty articles were sought for retrieval, eight of which were abstract-only articles. Forty-two full-text articles were assessed for eligibility. Twenty-seven articles were subsequently excluded, leaving the 15 articles included in this review (Figure 1). The PRISMA 2020 flow diagram detailing the identification and screening of articles is presented in Figure 1. The basic characteristics and details of the included pre-clinical and clinical articles are presented in Table 2 and Table 3, respectively. The quality assessment results using the JBI and NOS are presented in Appendix A.

### 3.1. Preclinical Studies

Four of the included studies were pre-clinical [70,71,72,73], and all were from the same group using newborn rabbits following seizure induction with kainic acid (KA) injection. Despite the studies being intervention-based, NIRS data from seizures were derived from the control groups treated with saline following KA injection in order to determine the “normal” pathological process during seizures in newborn rabbits. In each of the four studies, the NIRS parameters measured were HbO_2_, HHb, and HbT. Results were presented as the mean concentration change in NIRS parameters over time following KA injection. The trend in parameters was similar in all four studies. These studies did not distinguish between the pre-ictal, ictal, and post-ictal periods and only noted the overall trend in haemoglobin parameters following KA administration. All changes were reported in comparison with baseline values immediately prior to KA injection. Following KA injection, HbO_2_ initially decreased, and HHb initially increased [70,71,72,73]. HbT was shown to initially decrease in three studies [70,71,73]; however, one study described an initial rapid increase [72]. Following the initial decrease in HbO_2_, increases to baseline values were observed in all studies [70,71,72,73], with one increase surpassing baseline values before slowly decreasing towards the baseline [72]. HHb levels mostly decreased after reaching a maximum [70,71,73], with one returning to baseline [71] and the other two remaining above baseline [70,73]. Takei et al., 2001, however, demonstrated HHb levels continuing to increase following the initial KA injection [72]. HbT levels increased after reaching a minimum [70,71,73] to baseline [71] or beyond [70,73].

### 3.2. Clinical Studies

A total of 11 clinical studies were identified [43,46,74,75,76,77,78,79,80,81,82], of which eight were case reports [43,46,75,77,78,79,80,82] and three were cohort studies [74,76,81]. The studies reported on NIRS parameters, HbO_2_, HHb, HbT, HbD, rSO_2_, FTOE and CCO. In these studies, HIE was the most common underlying pathology. A variety of medications and EEG monitoring systems were used in these studies, which are summarised in Table 3.

#### 3.2.1. Changes in Individual Haemoglobin Parameters

**Figure 2:**
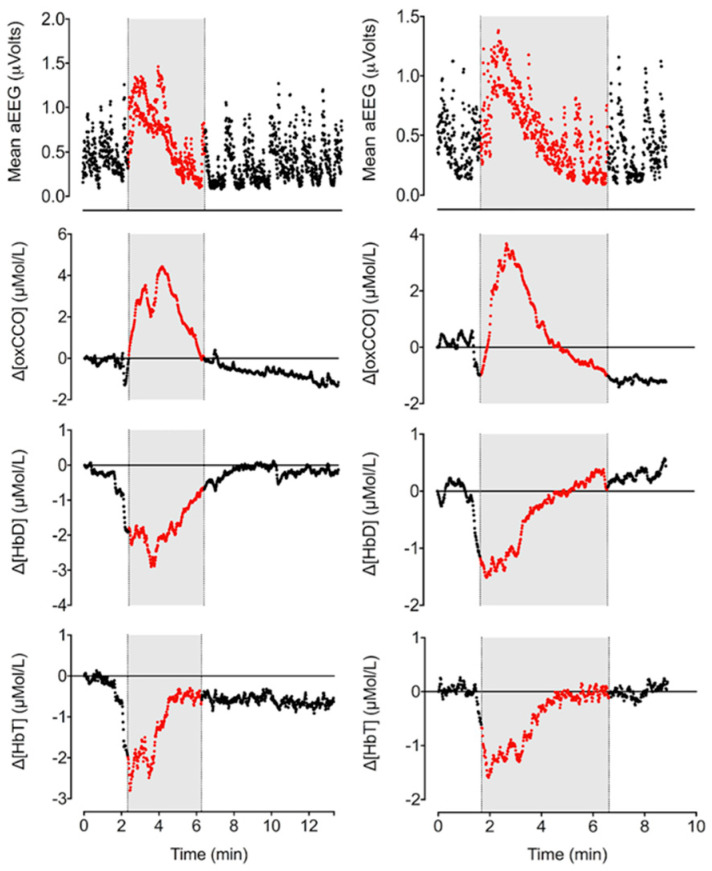
Changes in cerebral metabolism (oxCCO), oxygen delivery (HbD) and blood volume (CBV) during seizures in an infant with HIE (left = seizure 4 of 5, right = seizure 5 of 5). Seizure periods were identified on raw EEG and are marked here in grey [46] (reproduced with permission under the Creative Commons Attribution 4.0 International License (http://creativecommons.org/licenses/by/4.0/, accessed on 26 June 2022).

Pre-ictal changes in cerebral haemodynamics were reported in four studies [43,46,80,82]. Singh et al. [43,80] reported an increase in all haemoglobin parameters (HbO_2_, HHb and HbT) prior to the onset of seizures. These changes were reported as remarkably consistent between seizure events. A widespread increase in HbT was described 10 s prior to seizure onset using a diffuse optical tomography (DOT) imaging system [43,80]. Wallois et al. [82] also reported an initial increase in HbO_2_, HHb and HbT in all seizures except for five left-sided seizures where an initial drop in HbO_2_ was clearly observed with an increase in HHb. Focal right and left temporal lobe seizures were observed in this study. Mitra et al. documented the pre-ictal haemodynamic changes immediately before the seizure onset documented on raw EEG and noted a decrease in HbT and HbD (Figure 2) [46].

During the ictal phase, four studies reported changes in haemoglobin parameters [43,46,80,82]. Mitra et al. [46] presented the changes during individual seizures in detail in relation to electrical changes. This study recorded frontocentral changes on EEG with frontal optodes for BNIRS. In this study, a decrease in HbO_2_ occurred, whilst HHb increased during the ictal period. HbD also increased, demonstrating an increase in oxygen delivery to tissue. Singh et al. [43,80] reported that a maximum concentration of HbO_2_ and HHb was reached approximately 15 s after electrographic seizure onset. Following the maximum, a sharp decrease was observed, demonstrating a biphasic response. The decrease in HbO_2_ resulted in an increasingly lower baseline for each subsequent seizure due to the magnitude of the decrease in HbO_2_. Wallois et al. [82] also observed the same biphasic response with HbO_2_ and HHb. Singh et al. [43,80] reported specifically on two DOT channels over the left frontal lobe, demonstrating dramatically different responses in HbT, likely to be related to individual hemispheric pathology. In one channel during each seizure, HbT would dramatically increase and then return to baseline, but in the other HbT, levels would increase during the inter-ictal phase and rapidly decrease during the ictal phase. Wallois et al. [82] demonstrated an increase in HbT, whilst Mitra et al. [46] demonstrated a decrease, which could be related to different cerebral blood flow responses to seizures. Singh et al. [43,80] observed the changes in haemoglobin parameters to occur simultaneously with each other, whereas Wallois et al. [82] observed the parameter changes to occur sequentially. The sequence started with a prominent increase in HHb at 2.67 ± 1.28 s, followed by an increase in HbO_2_ at 4.22 ± 1.18 s and an increase in HbT at 3.82 ± 1.59 s when compared to the seizure onset of a “seizure-like activity” in a neonate with HIE. The delayed response time was thought to occur due to differing stimulus-response times in venules and capillaries.

**Figure 3:**
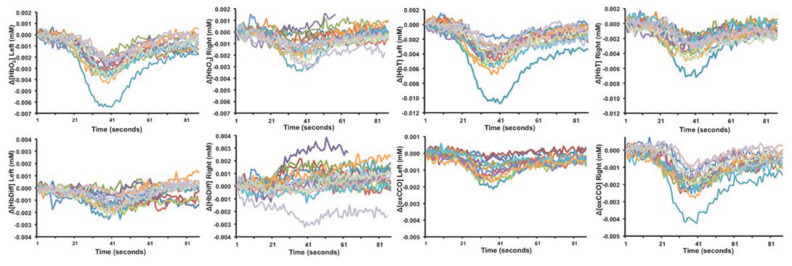
Transient haemodynamic and metabolic oscillatory changes in the postictal period following perinatal stroke [77]. Each colour indicates a separate event. Reproduced with permission under the Creative Commons Attribution 4.0 International License (http://creativecommons.org/licenses/by/4.0/) accessed 26 June 2022.

In the post-ictal period, a slow return towards baseline HbO_2_, HHb, HbT and HbD values was observed in all studies [43,46,80,82]. Two studies [46,74] demonstrated the occurrence of repetitive, transient oscillatory haemodynamic and metabolic changes with different NIRS devices (Figure 3). The electrical activity on EEG was suppressed during these periods. Transient changes exhibited a rapid decrease to a minimum and a slow recovery to baseline in optical markers and were more significant on the injured side [46]. A clear hemispheric difference was noted in cases of perinatal strokes in both studies. These changes were noted over an average duration of 90 s. No corresponding systemic changes were observed during these episodes. The aetiology of these events was unclear.

#### 3.2.2. Changes in Cerebral Oxygenation

Six clinical studies reported on changes in rSO_2_ during neonatal seizures [75,76,78,79,81,82]. Between studies, there were marked similarities in rSO_2_ trends. In the preictal period of a single study, rSO_2_ was noted to rise on the left side −1.58 ± 1.59 s prior to seizure onset during five out of ten subclinical seizures arising from the left temporal lobe, as recorded by direct current EEG and NIRS. The study did not report on rSO_2_ in the right side of the brain during this pre-ictal period [82]. Another study reported rSO_2_ rising before the onset of right frontal lobe subclinical EEG seizures when the seizure duration was greater than 100 s. The anatomical location of this rise in rSO_2_, however, was not specified, nor were the pre-ictal rSO_2_ changes in seizures lasting less than 100 s [78].

**Figure 4:**
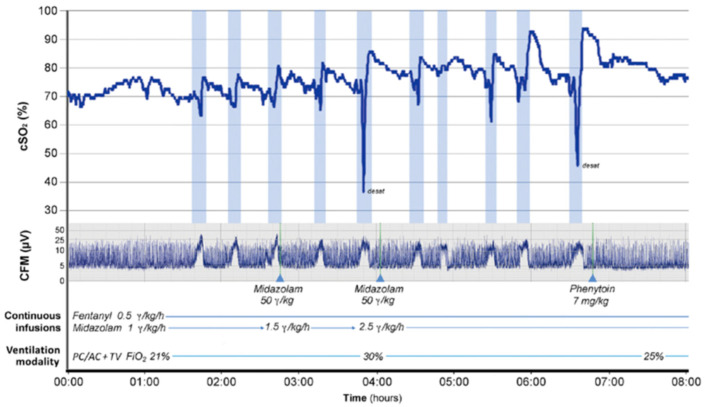
Changes in rSO_2_ and electrical activity on cerebral function monitoring (CFM) trace during seizures (highlighted by blue bars). SIPPV + VG is the ventilatory mode, synchronized intermittent positive pressure ventilation with volume guarantee [75]. Copyright (2019) by (Georg Thieme Verlag KG Stuttgart). Reproduced with permission of (Georg Thieme Verlag KG Stuttgart) in the format of (Journal/Magazine/Newspaper) via Copyright Clearance Center. Permission remains with (Georg Thieme Verlag KG Stuttgart). Any reuse of this image must be requested from the publisher directly.

During the ictal period, five out of the six studies reported a decrease in rSO_2_ in some or all seizures included in the studies [75,78,79,81,82]. Martini et al. [75] reported in detail the rSO_2_ changes that occurred in an infant with HIE exhibiting serial electroclinical seizures (Figure 4). Shortly after the onset of each individual seizure, a decrease in rSO_2_ was observed, followed by a rapid increase in rSO_2_ and then a gradual decline before seizure offset. This pattern of changes occurred within each observed seizure, suggesting a possible neurovascular coupling during neonatal seizures.

Wallois et al. [82] and Sokoloff et al. [81] reported that all seizures regardless of duration resulted in a decrease in rSO_2_, reflecting an increase in metabolic demand. The changes in rSO_2_ reported in these studies were observed in the seizure-originating hemisphere in HIE [82] and in both hemispheres in infants with a wide range of pathologies including HIE [81]. Other studies reported a mixed picture, with some seizures showing an increase and others a decrease in rSO_2_ [78,79]. Shuhaiber et al. [78] found that all subclinical seizures in the right frontal lobe in a neonate with tuberous sclerosis that lasted >100 s resulted in a decrease in rSO_2_ during or shortly after the seizure, but seizures <63 s did not show this pattern, suggesting that the metabolic demand during prolonged seizures could not be met, resulting in relative tissue hypoxia. The periods of relative tissue hypoxia (based on a drop in rSO_2_) during seizures were only observed in the right hemisphere, and rSO_2_ fluctuations were noted to be significantly larger than in the left hemisphere. Silas et al. [79] reported on an infant with HIE experiencing 13 subclinical seizures between postnatal 14–37 h of age. A larger rSO_2_ fluctuation in seizures >7 min (change of 7.8–28% of baseline rSO_2_) compared to <7 min (2.3–6.9%) was seen; however, only two out of seven seizures >7 min demonstrated a decrease in rSO_2_, with five out of seven demonstrating an increase in rSO_2_. Seizures < 7 min were not reported to have any associated rSO_2_ changes. When considering the seizures sequentially, the overall baseline in rSO_2_ appeared to decrease each time. The only substantial increase, from 63.0 to 75.6% in baseline, occurred when the inter-ictal period was 16 h. It appears as though this extended inter-ictal period acts as a recovery phase. Pacella et al. [76], however, reported that during all seizures in a neonate with HIE, a statistically significant increase in rSO_2_ occurred compared to inter-ictal periods.

Wallois et al. [82] commented on changes during the post-ictal period; rSO_2_ remained low and gradually recovered with time, as observed in 13 out of the 20 reported seizures. No further characteristics, however, were discussed.

#### 3.2.3. Metabolic Changes

Three papers reported on the metabolic changes during and after neonatal seizures [46,77,81]. FTOE, an indirect marker of metabolism, was reported by Sokoloff et al. [81]. Left- and right-sided FTOE were highest during seizures in all infants when compared to baseline and post-ictal values. These results suggest that the supply of oxygen may be insufficient to meet metabolic demands during neonatal seizures. Concentrations of CCO during and after neonatal seizures, a direct marker of metabolism, were reported by Mitra et al. [46,77]. Following seizure onset, oxCCO increased up to a maximum, reflecting an increase in mitochondrial oxidative metabolism. Once the peak on aEEG had been reached, oxCCO began to fall (Figure 2). Beyond seizure offset, oxCCO continued to fall, leading to a progressively lower baseline [46]. The progressive decrease in oxCCO baseline with sequential seizures may indicate reduced mitochondrial oxidative metabolism.

## 4. Discussion

The findings from this systematic review provide important contributions to our understanding of the role of optical monitoring for neonatal seizures. Clear haemodynamic and metabolic changes were described using optical monitoring during pre-ictal, ictal, and post-ictal periods.

During seizures, neuronal energy demand increases. Neurons require a continuous supply of glucose and oxygen for neuronal communication due to their inability to store energy [83]. The increased metabolic rate during seizures has been demonstrated in previous adult animal studies [84,85,86]. Ictal changes in oxCCO concentrations during seizures give a clear indication of changes in mitochondrial oxidative metabolism [46]. As the metabolic rate increases, the rate of ATP production increases, and so does the concentration of oxCCO to meet the energy demand, hence the initial lag before oxCCO increases following seizure onset. Previous studies have demonstrated a decline in high-energy phosphate stores during seizures [45,87], corresponding to the changes in mitochondrial activity [61,88]. The findings of this review correlate with what was previously known about cerebral energy metabolism during neonatal seizures. A rapid increase in [oxCCO] occurring at the onset of seizures [62] or in BNIRS channels where seizures were initiated in a paediatric population was also demonstrated [89]. A greater proportion of ATP is generated through non-oxidative metabolism in the preterm brain compared to that at term [87,90]. It could, therefore, be hypothesised that changes in [oxCCO] may differ during neonatal seizures, depending on gestational age. Recommendations would, therefore, involve analysing future research in categories of gestational age to determine whether there are any differences in metabolism during neonatal seizures in preterm and term neonates.

In the healthy brain, changes in local haemodynamic parameters are closely related to the metabolic demands of neurons. Whether this relationship is maintained during seizures remains controversial and has been studied extensively in newborns, adults and animal models using functional MRI (fMRI) [91] and optical methods. It has been demonstrated that seizures can cause alterations in neonatal cerebrovascular function. Changes in haemodynamic parameters, such as cerebral blood flow, were observed in epileptic foci and other brain regions minutes before spontaneous seizures by NIRS and MRI [92].

Wallois et al. [93] previously summarised the different types of haemodynamic responses seen in participants with epilepsy from various studies. This demonstrated a lack of consistency between HbO_2_, HHb, and HbT changes during seizures, which is consistent with the findings from this systematic review. The lack of consistency is likely to result from differences in age at onset of seizures, seizure type, the position of the seizure focus, background aetiology, severity of the background brain injury, optical instruments used for monitoring and optode placement. Studies have demonstrated that during electrographic seizures HbO_2_, HHb and HbT decreased; however, during electroclinical seizures, HbO_2_, HHb and HbT increased [94]. Despite the increasing metabolic rate [94], human and animal studies have demonstrated that absence seizures are characterised by a reduction in CBF [95,96,97,98]. Complex partial and generalised seizures, however, have been characterised by an increase in CBF and metabolic rate [99,100,101,102]. Differing background aetiologies and injury severity, in particular, will impact ictal changes in CBF and CBV, with injury severity also impacting mitochondrial function. Changes in CBF are a key factor in understanding ictal pathophysiological changes in real-time. Boylan et al. [103] demonstrated an increased cerebral blood flow velocity during seizures using transcranial doppler ultrasound. Recent advances in optical technologies present an opportunity to monitor microvascular CBF continuously using diffuse correlation spectroscopy [104]. Injury severity will likely have an impact on CBF response during seizures, and regional differences in CBF response in relation to seizure focus will produce different degrees of haemodynamic impact in different areas of the brain.

Interestingly, despite differing haemodynamic patterns between studies, recurrent seizures in participants within the same study have shown remarkable consistencies [43,46,74,80]. Such findings could indicate that biomarker changes are unique to the individual based on age, seizure type and focus, background aetiology and injury severity. Differences in the post-ictal hemispheric changes were clear following perinatal stroke. Regional differences in cerebral haemodynamics and metabolism are likely to be related to seizure foci [105].

Some studies have demonstrated that during seizures, HbT increases by approximately 10% [106], with others demonstrating a decrease [92,107]. Considering metabolic changes, HbO_2_ would be expected to undergo an initial decrease [92,106], before increasing as cerebral perfusion increases. Potential explanations for the variety of changes noted may be due to the timing and resolution of the measurement presented in the studies. Whilst some studies recorded and reported on continuous NIRS measurements, others reported mean changes that may, therefore, overlook more subtle changes in parameters. The resolution of the captured data varies between different NIRS systems, which further contributes to perceived differences in the optical markers. Depending on the initial focus of the seizure, results in parameter changes detected may also differ based on sensor location. Without comparing the haemodynamic biomarker results to other physiological variables, it is impossible to determine whether these changes are localised to the brain or are occurring due to systemic changes in blood pressure [108]. All future studies reporting cerebral changes should ideally present the relevant changes in physiological variables.

The biomarker with the most consistent pattern appears to be HHb, even though fluctuations are not always significant. In the included studies, HHb increased during the pre-ictal or ictal phase of neonatal seizures [43,46,74,80,82], and one study reported that the change was not statistically significant. This is consistent with previous studies that demonstrated that seizures induce an increase in HHb, which is immediately followed by an increase in HbO2 and HbT [109], or no consistent change in HHb [92], regardless of the changes occurring in HbO2 and HbT [110]. When considering the increase in metabolic activity during neonatal seizures and the decrease in cerebral oxygenation, the increase in HHb would be expected during the relative tissue hypoxia before the increase in cerebral perfusion. Cerebral blood flow has been shown to transiently increase in over 90% of seizures [108]. The degree to which HHb increases may be due to seizure duration, the extent of underlying brain damage, or the degree of tissue hypoxia.

Trends in rSO_2_ levels vary during neonatal seizures. In some studies, baseline values demonstrated an overall downtrend in baseline [79], whereas others reported an overall upward trend [75]. During each seizure, however, there was a drop in rSO_2_ [75,79,81], suggesting an increase in metabolic activity and confirming the significant rSO_2_ fluctuations described during seizures [111]. In paediatric traumatic brain injury, studies have demonstrated a progressive decline in rSO_2_ values in recurring seizures [94].

In the included studies, six different NIRS devices were used. Whilst all NIRS devices operate under the same fundamental principles, each signal-processing algorithm is proprietary in nature and, therefore, limits the ability to compare absolute values between devices [57]. Direct analysis of several available commercial devices has been performed [112,113,114,115], with results demonstrating that despite algorithm differences, a strong correlation between the devices was observed, rendering them largely equivalent [57]. Mathematical models have, however, been developed to correct absolute measurements between devices used [116,117,118]. Aside from the device itself, it is important to consider the sensors used in neonates. The majority of commercial NIRS monitors have specific paediatric or neonatal sensors [57]. Beyond the physical size differences, the neonatal probes have shorter distances between the emitting light source and detector. These distances allow near-infrared light to penetrate the tissues at different depths through the skull [119]. Whilst the overall trend in NIRS parameters should not change, studies have demonstrated that different types of sensors can produce differences in absolute rSO_2_ values of up to 14% [120], and reapplication of the same sensor in the same position can alter absolute values by up to 6% [121]. The positioning of the sensors must also be considered to localise changes in optical parameters. As demonstrated by the results of this review, seizure location matters, and parameter changes can occur in different regions of the brain. It would, therefore, be important to monitor multiple locations in future studies of neonatal seizures to determine if regional changes are related to aetiology.

A recent study in adult epilepsy patients demonstrated a potential use for NIRS based on convolutional neural network applications as a predictor for seizures [122]. The study demonstrated the accuracy of HbO_2_ + HHb readings within a 5- to 2-min window before seizure onset, ranging between 96.9% and 100%, with sensitivity between 95.24% and 100% and specificity between 98.57% and 100%. These data are supported by the findings from this review that changes in metabolic and haemodynamic parameters can be visualised prior to the onset of neonatal seizures. If further larger studies replicated these findings, there is the potential for this technology to be extended into neonatal care. Such a predictor tool may be invaluable to administer early treatments and subsequently reduce the associated morbidity and mortality rates. Another recent study identified the epileptic seizure focus in a child with focal cortical dysplasia using a BNIRS system combined with video-EEG [109], promising a possible use of optical systems for the prediction and identification of seizures.

Both NIRS and EEG can record brain function with high temporal resolution of the neuronal and haemodynamic/metabolic components of brain function. These two neuromonitoring modalities are complementary and provide a more complete picture of the different intricate relationships of brain function. Near-infrared light does not interfere with the electrical EEG measurement. The option of multimodal EEG-NIRS monitoring allows the macroscopic temporal dynamics of brain electrical activity and spectral measurements of haemodynamic fluctuations to be analysed independently and simultaneously. Simultaneous use is most valuable during the study of neonatal seizures to establish the relationship between signals during activation. Such technology at the bedside, however, not only requires specialists to interpret the EEG data but presents challenges with respect to sensor positioning, identification of artefacts and synchronising the two devices [123].

## 5. Limitations

The key limitations in the review process were the lack of randomised controlled trials in this field and the limited sample sizes. Several studies included in this review were considered to be of poor quality according to the NOS due to the lack of follow-up of the infants. Given that the data extracted from the included cohort studies were observational and not intervention-based, the lack of such data is not likely to compromise the quality of the results presented. Neonates included in these studies were medically treated through a variety of means, including therapeutic hypothermia and various anti-seizure medications, as well as possible inotropic support, all of which also have an impact on cerebral physiology. In neonates where anti-seizure medication was used, a variety of medications were often used consecutively in an attempt to terminate the seizure, meaning the effect of this medication on the haemodynamic changes visualised could not be determined. Over the past few decades, there have been no new therapies for neonatal seizures. It is now essential to understand the background changes in further detail and develop essential cot-side biomarkers that may aid clinical practice as well as future trials in this area.

One criticism regarding the methodology of the included studies is the limited number of optodes used during NIRS. Whilst placing detectors on the forehead reduces the effect of hair on NIRS measurements, it may lead to an increased distance between the seizure site and the detector. Such situations may lead to reports of only regional haemodynamic or metabolic changes during seizures, as studies have demonstrated a potentially different response that may occur at the peripheral seizure site [84]. Not all studies reported the type or location of seizures in the brain, meaning this review was unable to correlate any specific haemodynamic or metabolic changes based on these factors. Whilst all studies included in this review reported on trends in haemodynamic and metabolic changes that occurred during seizures, not all studies used continuous NIRS monitoring. Slight changes in parameters or rapid changes may, therefore, not be truly reflected during a seizure if measurements were taken at specific time intervals.

One other key limitation in the studies was the lack of detailed descriptions of individual EEG-specific changes during seizures and how those correlated with respective changes in NIRS variables.

## 6. Future Direction

Clinicians are becoming increasingly aware that a “one size fits all approach” does not allow care to be optimised for all patients. One challenge, therefore, arising with NIRS technology is the ability to interpret such information quickly and easily at the bedside [124]. Whilst trends in NIRS parameters are a rich physiological information source [125,126], the use of cerebral oxygenation as the key predictor of changes in clinical practice may be challenging due to the wide range of variables that have the potential to influence such readings. BNIRS has the potential to overcome such challenges in a clinical setting due to its ability to directly measure metabolic changes in the neonatal brain [62] along with changes in cerebral oxygenation. Whilst its applications have not yet been investigated widely, it is a highly accurate neuromonitoring tool with the ability to detect seizures [127]. It has the potential to be used independently of traditional monitors to improve the practicalities of real-time monitoring without compromising the sensitivity and specificity of seizure detection in neonates. Whilst EEG is considered the current gold-standard, it requires the involvement of specialists or extensive training for providers, meaning the logistics of interpreting the recording 24 h a day, 7 days a week are massive, and most neonatal units do not have access to such equipment and/or the support for continuous neurophysiology review of EEG monitoring [128]. aEEG is often used in place of EEG; however, its simplified nature for ease of review by the clinician results in lower sensitivity and specificity for detection of seizures, in particular, those that are of low amplitude, brief, or distant from the EEG leads [129,130].

The deep understanding that NIRS can provide data on the metabolic impact of seizures can help us determine which seizures are harmful to the neonatal brain and which are not. Further studies investigating the role of CCO as a biomarker for neonatal seizures and outcome prediction are, therefore, desirable. BNIRS, together with diffuse correlation spectroscopy, has further potential to present a clearer picture regarding the pathophysiological changes associated with seizures in real-time in the neonatal intensive care unit. This will further elucidate the details of changes in neurovascular and flow-metabolism coupling in the peri-ictal period and might be an important marker for seizure detection with or without EEG. Given the lack of evidence regarding the efficacy of many antiseizure medications in the neonatal period, it is essential to review the impact of these medications on seizure-induced changes in cerebral haemodynamics and metabolism. The optical markers of seizures need to be assessed in future studies for an in-depth assessment of the impact of common anti-seizure medications currently used and to be trialled in future.

We suggest future studies in this area to detail individual seizure-specific changes on EEG, particularly the origin (focal and generalised) and migration. It would be useful to describe the respective optical changes in different types of seizures.

## 7. Conclusions

Common patterns of detectable haemodynamic and metabolic changes can be monitored at the cot side using NIRS and other optical technologies. The substantial advancement in optical technology over the past few decades presents a unique opportunity to improve our current understanding of pathophysiological changes in neonatal seizures. The variability in reported responses in optical markers is likely to be related to aetiology and severity of the underlying brain injury, CBF response, type of seizure, as well as the regional differences in relation to distance from seizure origin and placement of optodes for monitoring. Larger studies are necessary to understand these differences and establish optical monitoring in clinical practice to guide the management and prognostic assessment of neonatal seizures.

## Figures and Tables

**Table 1:** Search strategy used for Medline (Ovid version) search.

1	(brain metabolism) or (brain function) or (tissue metabolism) or ([cerebr* adj3 (metabol* or oxygenation or haemodynamic or haemodynamic or volume or blood flow)]) or (near infrared spectroscopy) or (near infrared adj3 spectroscop*) or (exp Spectroscopy, Near-Infrared/) or (NIRS) or (broadband near-infrared spectroscopy) or (broadband NIRS) or (exp Tomography, Optical/) or (exp Optical Imaging/) mp. [mp = title, abstract, original title, name of substance word, subject heading word, floating sub-heading word, keyword heading word, organism supplementary concept word, protocol supplementary concept word, rare disease supplementary concept word, unique identifier, synonyms] Results = 163,109
2	(newborn) or (baby) or (babies) or (infan*) or (neonat*) mp. [mp = title, abstract, original title, name of substance word, subject heading word, floating sub-heading word, keyword heading word, organism supplementary concept word, protocol supplementary concept word, rare disease supplementary concept word, unique identifier, synonyms] Results = 1,614,827
3	(exp Seizures/) or (seizure*) mp. [mp = title, abstract, original title, name of substance word, subject heading word, floating sub-heading word, keyword heading word, organism supplementary concept word, protocol supplementary concept word, rare disease supplementary concept word, unique identifier, synonyms] Results = 160,257
4	1 and 2 and 3 Results = 446
5	Limit 4 to English language Results = 413

**Table 2:** Preclinical data extracted from the included articles.

Findings	Pre-Ictal, Ictal, or Post-Ictal Data	NIRS Biomarkers	NIRS Device, Sensor Positioning	Seizure Induction Agent	Participants (Number, Median Age, Species)	Study Design	First Author, Year
Following KA administration, HbO_2_ and HbT decreased at the same time as an increase in HHb. The HbO_2_, levels then gradually returned to baseline levels before KA administration. HHb levels, however, continued to increase. Once the HbT levels reached a minimum, they increased gradually beyond baseline values.	pre-ictal, ictal, and post-ictal	HbO_2_, HHb, HbT	NIR 1000	Kainic Acid	6 2 weeks Rabbit	Cohort Study	Takei et al., 1999 [70]
Following KA administration, HbO_2_ and HbT decreased, followed by an increase in HHb. The HbO_2_, HbT and HHb levels then gradually returned to baseline levels prior to KA administration.	pre-ictal, ictal, and post-ictal	HbO_2_, HHb, HbT	NIR 1000	Kainic Acid	6 2 weeks Rabbit	Cohort Study	Takei et al., 1999 [71]
During KA-induced seizures, HbO_2_ was shown to decrease initially to a minimum, then increase beyond baseline values before slowly decreasing to baseline. HHb was shown to increase during seizures. HbT slowly increased to a maximum before decreasing.	pre-ictal, ictal, and post-ictal	HbO_2_, HHb, HbT	NIR 1000	Kainic Acid	6 2 weeks Rabbit	Cohort Study	Takei et al., 2001 [72]
HbO_2_ and HbT initially decreased following KA administration. HbO_2_ then slowly increased towards baseline values. HbT increased beyond baseline values. HHb initially increased before starting to decrease and plateau.	pre-ictal, ictal, and post-ictal	HbO_2_, HHb, HbT	NIR 1000	Kainic Acid	6 2 weeks Rabbit	Cohort Study	Takei et al., 2002 [73]

**Table 3:** Clinical data extracted from the included articles.

Findings	Pre-Ictal, Ictal, or Post-Ictal Data	NIRS Biomarkers	NIRS Device, Sensor Positioning	Type of EEG Monitoring Used	Treatments Used	Underlying Pathology in Neonate(s)	Participants (Number with NIRS Data, Median Gestational Age, Species)	Study Design	First Author, Year
During each event, HbO_2_, HHb and HbT increased to a maximum. They then rapidly decreased to a minimum before slowly returning to baseline values. HHb changes were generally not significant.	Post-ictal	HbO_2_, HHb, HbT	UCL Diffuse Optical Tomography, Cap	Full EEG-OT with clinical, 9-electrode EEG montage or reduced-electrode EEG	Phenobarbital, phenytoin, clonazepam and midazolam infusion.	Severe neonatal HIE following right temporal haemorrhagic stroke, Left middle and posterior cerebral artery infarct, suspected sepsis at birth, hypoglycaemia.	4 40 weeks Human	Cohort Study	Cooper et al., 2011 [74]
Rhythmic rSO_2_ fluctuations occurred during each seizure. Within each episode, rSO_2_ levels initially decreased, followed by a sharp increase and a slow decrease toward baseline. Baseline rSO_2_ levels increased with each subsequent seizure.	Pre-ictal, ictal, and post-ictal	rSO_2_	-	aEEG	Therapeutic hypothermia, midazolam, phenobarbital, and phenytoin	HIE	1 35 weeks Human	Case Report	Martini et al., 2019 [75]
Regardless of outcome, rSO_2_ values during seizures trended towards being significantly higher than periods without seizures (85% vs. 81%, *p*= 0.0534).	Ictal	rSO_2_	INVOS, Left or right side of the forehead	vEEG	Therapeutic hypothermia	HIE	1 38 weeks Human	Case Report	Pacella et al., 2020 [76]
At the start of each seizure, CCO increased. Following the aEEG peak, CCO fell to below baseline levels, leading to a progressively lower baseline with each subsequent seizure. HbT and HbD both decreased initially and then slowly returned to baseline after the aEEG peak.	Ictal, post-ictal	CCO, HbT, HbD, HbO_2_, HHb	Broadband NIRS, Both sides of the forehead, using an optode distance of 2.5 cm	10-channel neonatal EEG (Nicolet EEG monitor), aEEG trends were derived	Therapeutic hypothermia, phenobarbitone, ionotropic support	HIE	1 38 weeks Human	Case Report	Mitra et al., 2016 [46]
During the post-ictal period, synchronous and repeater transiter changes were noted in HbO_2_, HHb and CCO in both hemispheres. The parameters acutely dropped before a slow increase toward baseline. This pattern of changes occurred in 16 separate events.	Post-ictal	CCO, HbT, HbD, HbO_2_, HHb	Broadband NIRS One NIRS channel was placed on either side of the forehead. Four detected optodes were placed horizontally against each source optode with source-detector distances of 1.0, 1.5, 2.0 and 2.5 cm.	Nicolet EEG monitor	Phenobarbitone, phenytoin, midazolam, paraldehyde	Neonatal Stroke	1 40 weeks Human	Case Report	Mitra et al., 2016 [77]
Every seizure lasting longer than 100 s occurred as rSO_2_ levels were rising and was associated with a drop in rSO_2_ levels.	Ictal	rSO_2_	INVOS 5100, Light sources placed 2 cm above the eyebrows and 2 cm from the midline on the right and left sides of the forehead. Detectors were placed at the same level in front of the ears.	vEEG	Phenobarbital, fosphenytoin	Tuberous sclerosis	1 38 weeks Human	Cohort Study	Shuhaiber et al., 2004 [78]
rSO_2_ changes occurred in 7 out of 8 seizures that lasted 7 min or longer. 4 out of 5 seizures that lasted less than 7 min were not associated with any rSO_2_ changes.	Ictal	rSO_2_	NIRO 200, Right frontal parietal region and covered with light-proof cloth.	Two-channel aEEG with electrodes at F3-P3, F4-P4 positions	Therapeutic hypothermia, phenobarbitone, phenytoin and midazolam infusion	HIE	1 39 weeks Human	Cohort Study	Silas et al., 2012 [79]
30 s prior to seizure onset, an increase in HHb, HbO_2_ and HbT was observed. After reaching a maximum, all three parameters decreased until they reached a minimum. Concentrations then began to recover toward baseline levels.	Pre-ictal, ictal, and post-ictal	HHb, HbT, HbO_2_	UCL Diffuse Optical Tomography, Cap	aEEG, Dot-EEG with flexible head cap	Therapeutic hypothermia, phenobarbital, clonazepam, and phenytoin.	Severe HIE	1 40 weeks Human	Case Report	Singh et al., 2014 [43]
Prior to electrographic onset, an increase in HbO_2_, HHb and HbT was observed to a maximum level. All three parameters began to decrease prior to electrographic offset. The extended decrease occurred, and parameters recovered to baseline values.	Pre-ictal, ictal, and post-ictal	HbO_2_, HHb, HbT	UCL Diffuse Optical Tomography, Cap	DOT-EEG (standard 11-channel EEG neonatal montage)	Therapeutic hypothermia,	Severe HIE	1 40 weeks Human	Case Report	Singh et al., 2016 [80]
rSO_2_ levels declined during seizures in both hemispheres. FTOE increased during seizures.	Ictal	rSO_2_, FTOE	CYtochrome Research Instrument and appLication (CYRIL), Both sides of the forehead, using an optode distance of 2.5 cm	vEEG	Phenobarbital	HIE, neonatal epilepsy syndromes, arterial ischaemic stroke, sepsis, benign neonatal seizures, sinovenous thrombosis, intracranial haemorrhage, and uncertain	20 39 weeks Human	Cohort Study	Sokoloff et al., 2015 [81]
Seizures exhibited biphasic changes. HHb initially increased, then decreased, which was associated with an increase in HbO_2_ and HbT. During seizures, rSO_2_ always decreased slightly.	Pre-ictal, ictal	HbO_2_, HHb, HbT, rSO_2_, FTOE	Imagent from ISS, Cap	High-resolution direct-current (HR DC) EEG and Alternating-current electroencephalography (AC EEG) with electrode cap	Phenobarbital	Acute foetal distress resulting in caesarean section	1 40 weeks Human	Case Report	Wallois et al., 2009 [82]

## Data Availability

Not applicable.

## Appendix A

**Table A1:** Newcastle-Ottawa Scale appraisal of included cohort studies.

Author	Title	Year	Selection Bias Assessment (Maximum 4 Stars)								Comparability (Maximum 2 Stars)		Outcome (Maximum 3 Stars)						Total Score	Quality
			Representativeness of the Exposed Cohort		Selection of the Non-Exposed Cohort		Ascertainment of Exposure		Demonstration That Outcome of Interest Was Not Present at Start of Study		Comparability of Cohorts on the Basis of the Design or Analysis Controlled for Confounders		Assessment of Outcome		Was Follow-Up Long Enough for Outcomes to Occur		Adequacy of Follow-Up of Cohorts			
			Selection	Score	Selection	Score	Selection	Score	Selection	Score	Selection	Score	Selection	Score	Selection	Score	Selection	Score		
Singh et al. [80]	Neurovascular interactions in the neurologically compromised neonatal brain	2016	b	1	c	0	a	1	b	0	b	1	d	0	b	0	a	1	4	Poor
Sokoloff et al. [81]	Phenobarbital and neonatal seizures affect cerebral oxygen metabolism: a near-infrared spectroscopy study	2015	b	1	c	0	a	1	a	1	b	1	d	0	b	0	a	1	5	Poor
Cooper et al. [74]	Transient haemodynamic events in neurologically compromised infants: A simultaneous EEG and diffuse optical imaging study	2011	b	1	c	0	a	1	b	0	b	1	d	0	b	0	a	1	4	Poor
Takei et al. [72]	Different effects between 7-nitroindazole and L-NAME on cerebral hemodynamics and hippocampal lesions during kainic acid-induced seizures in newborn rabbits	2001	b	1	a	1	a	1	a	1	b	1	d	0	b	0	a	1	6	Poor
Takei et al. [71]	Effects of nitric oxide synthase inhibition on changes in cerebral oxygenation and cerebral blood flow during kainic acid induced seizures in newborn rabbits	1999	b	1	a	1	a	1	b	0	b	1	d	0	b	0	a	1	5	Poor
Takei et al. [70]	Effects of nitric oxide synthase inhibition on the cerebral circulation and brain damage during kainic acid-induced seizures in newborn rabbits	1999	b	1	a	1	a	1	a	1	b	1	d	0	b	0	a	1	6	Poor
Takei et al. [73]	Nitric oxide donor increases cerebral blood flow and oxygenation during kainic acid-induced seizures in newborn rabbits	2002	b	1	a	1	a	1	b	0	b	1	d	0	b	0	a	1	5	Poor

**Table A2:** Joanna-Briggs Institute appraisal of included case reports.

Author	Title	Year	Were Patient’s Demographic Characteristics Clearly Described?	Was the Patient’s History Clearly Described and Presented as a Timeline?	Was the Current Clinical Condition of the Patient on Presentation Clearly Described?	Were Diagnostic Tests or Assessment Methods and the Results Clearly Described?	Was the Intervention(s) or Treatment Procedure(s) Clearly Described?	Was the Post-Intervention Clinical Condition Clearly Described?	Were Adverse Events (harms) or Unanticipated Events Identified and Described?	Does the Case Report Provide Takeaway Lessons?	Overall Appraisal
Martini et al. [75]	Cerebral Oxygenation Patterns during Electroclinical Neonatal Seizures	2019	✓	✓	✓	✓	✓	No	Not Applicable	✓	Include
Mitra et al. [46]	Changes in Cerebral oxidative Metabolism during Neonatal seizures Following Hypoxic–Ischemic Brain Injury	2016	✓	✓	✓	✓	✓	✓	Not Applicable	✓	Include
Mitra et al. [77]	In vivo measurement of cerebral mitochondrial metabolism using broadband near infrard spectroscopy following neonatal stroke	2016	✓	✓	✓	✓	✓	✓	Not Applicable	✓	Include
Shuhaiber et al. [78]	Cerebral regional oxygen fluctuations and decline during clinically silent focal electroencephalographic seizures in a neonate	2003	✓	✓	×	✓	✓	×	Not Applicable	✓	Include
Silas et al. [79]	Cerebral oxygenation during subclinical seizures in neonatal hypoxic-ischaemic encephalopathy	2012	✓	✓	×	×	✓	✓	Not Applicable	✓	Include
Singh et al. [43]	Mapping cortical haemodynamics during neonatal seizures using diffuse optical tomography: A case study	2014	✓	✓	✓	✓	✓	×	Not Applicable	✓	Include
Wallois et al. [82]	Haemodynamic changes during seizure-like activity in a neonate: A simultaneous AC EEG-SPIR and high-resolution DC EEG recording	2009	✓	✓	✓	✓	✓	✓	Not Applicable	✓	Include
